# Genetic Analysis of Mitochondrial Ribosomal Proteins and Cognitive Aging in Postmenopausal Women

**DOI:** 10.3389/fgene.2017.00127

**Published:** 2017-09-21

**Authors:** Khyobeni Mozhui, Beverly M. Snively, Stephen R. Rapp, Robert B. Wallace, Robert W. Williams, Karen C. Johnson

**Affiliations:** ^1^Department of Preventive Medicine, University of Tennessee Health Science Center Memphis, TN, United States; ^2^Department of Genetics, Genomics and Informatics, University of Tennessee Health Science Center Memphis, TN, United States; ^3^Department of Biostatistical Sciences, Wake Forest University School of Medicine Winston-Salem, NC, United States; ^4^Department of Psychiatry and Behavioral Medicine, Wake Forest University School of Medicine Winston-Salem, NC, United States; ^5^Department of Epidemiology, University of Iowa College of Public Health Iowa City, IA, United States

**Keywords:** gene-set analysis, cognitive aging, mitochondrial ribosomal proteins, APOE, lifespan

## Abstract

Genes encoding mitochondrial ribosomal proteins (*MRPs*) have been linked to aging and longevity in model organisms (i.e., mice, *Caenorhabditis elegans*). Here we evaluated if the *MRPs* have conserved effects on aging traits in humans. We utilized data from 4,504 participants of the Women's Health Initiative Memory Study (WHIMS) who had both longitudinal cognitive data and genetic data. Two aging phenotypes were considered: (1) gross lifespan (time to all-cause mortality), and (2) cognitive aging (longitudinal rate of change in modified mini-mental state scores). We tested genetic association with variants in 78 members of the *MRP* gene family. Genetic association tests were done at the single nucleotide polymorphism (SNP) level, and at gene-set level using two distinct procedures (GATES and MAGMA). We included SNPs in *APOE* and adjusted the tests for the *APOE*-ε4 allele, a known risk factor for dementia. The strongest association signal is for the known cognitive aging SNP, rs429358, in *APOE* (*p*-value = 5 × 10^−28^ for cognitive aging; *p*-value = 0.03 for survival). We found no significant association between the *MRPs* and survival time. For cognitive aging, we detected SNP level association for rs189661478 in *MRPL23* (*p*-value < 9 × 10^−6^). Furthermore, the gene-set analysis showed modest but significant association between the *MRP* family and cognitive aging. In conclusion, our results indicate a potential pathway-level association between the *MRPs* and cognitive aging that is independent of the *APOE* locus. We however did not detect association between the *MRP*s and lifespan.

## Introduction

Aging is a complex biological process that is characterized by overall decline in health, vigor, cognition, and increased vulnerability to numerous diseases. In addition to lifestyle and environmental factors, molecular pathways and cellular processes that are conserved across species may contribute to aging (Kenyon, 2010). Mitochondrial function has been central to aging research and dysfunctions in this organelle have been implicated in lifespan regulation, cognitive aging, and Alzheimer's disease (Bishop et al., 2010; Swerdlow, 2011; Picard and McEwen, 2014). The mitochondrion is a site of protein synthesis and studies in mice and *Caenorhabditis elegan*s (*C. elegans*) indicate that imbalances in protein translation between mitochondrial and nuclear genes can trigger life-extending signaling pathways (Houtkooper et al., 2013; Mouchiroud et al., 2013). Protein synthesis in mitochondria is exquisitely regulated by a translational machinery that is attuned to nuclear protein turnover (Poyton and McEwen, 1996). Components of the translational system, including the mitochondrial ribosomal proteins (MRPs), are encoded by the nuclear genome and subsequently translocated into the mitochondria. Mutations in the *Mrp* genes that reduce gene expression have a conserved life-extending effect in both mice and *C. elegans* (Houtkooper et al., 2013; Mouchiroud et al., 2013). In this study we evaluate if this effect seen in experimental settings in model organisms translates to aging and lifespan traits in humans.

Lifespan and mortality, while related to aging, are not definitive measures of aging and can only capture a gross outcome. There are many other phenotypes that define the health, vigor, and functional fitness of individuals as they age. The genetic and phenotypic heterogeneity partly explains why only a few gene variants have been consistently associated with aging. An exceptional case is *APOE*, which has shown replicable association with human longevity (Schachter et al., 1994; Nebel et al., 2011). The *APOE-*ε*4* risk allele is also consistently linked to cognitive decline, particularly in the context of pathological dementia and Alzheimer's disease (Chartier-Harlin et al., 1994; de Jager et al., 2012; Davies et al., 2014). Aside from a few examples like *APOE*, most genetic modulators of aging appear to have only small effects and are likely to mediate influence via complex gene networks and pathways (Yashin et al., 2010; Walter et al., 2011; Deelen et al., 2013).

Here we use the extensive resources from the Women's Health Initiative Memory Study (WHIMS) to test the collective effect of the *MRP* genes on two aging-related traits: gross survival time, and cognitive aging as a specific indicator of brain aging. Since *APOE* has an established effect, we included *APOE* variants in our analysis and evaluated effect of *MRPs* independent of *APOE*. We performed standard SNP level association tests followed by a pathway level gene-set test to examine the summarized effect of multiple variants within the *MRP* family.

## Methods

### Description of WHIMS study cohort

The multi-center WHI study was launched in 1993 (https://www.whi.org). Participants were postmenopausal women, between 50 and 79 years of age at time of enrollment. Women were recruited from 40 clinical centers in the United States and represent a diverse population. All participants provided written informed consent and all sites received IRB approval. Demographic and health characteristics, and numerous other measures were collected at baseline and follow-up visits and are detailed in WHI publications (Anderson et al., 2003; Hays et al., 2003). WHI had two major parts: a clinical trial and an observational study. The randomized clinical trial arm included a hormone therapy (HT) study that assigned women to estrogen-alone (E-alone intervention), estrogen and progesterone (E+P intervention), or placebo control groups. WHIMS is an ancillary study to the HT trial and all the women in WHIMS were drawn from the HT component of WHI and the majority (>89%) were Caucasians (Shumaker et al., 1998; Rapp et al., 2003). WHIMS started in 1996 and recruited 7,479 women. Participants for WHIMS underwent additional screening and were 65 years or older and were required to be free of dementia at enrollment. Participants were given annual cognitive tests during clinical visits. The present work is a secondary analysis on a subset of 4,504 WHIMS Caucasian participants. These 4,504 women are the subset of WHIMS that have genome-wide association study (GWAS) data and were all genotyped on the Illumina HumanOmniExpressExome-8 v1.0 Beadchips.

### Defining aging phenotypes

The first aging trait is the gross outcome: all-cause mortality or survival time. This was measured as days from enrollment to participant's uncensored death, the last National Death Index date, or censored at end-of-follow up (Seguin et al., 2014). The second aging trait was cognitive aging estimated by the rate of change in global cognitive function. We used cognitive decline rate as a proxy of age related functional decline rather than categorical classification into dementia cases vs. healthy controls. The Modified Mini-Mental State Examination (3MSE) was administered at time of screening (visit year 0) and annually for up to 11 years of follow-up visits. This score ranges from 0 to 100 with higher scores indicating higher global cognitive ability (Teng and Chui, 1987).

### Statistical analysis of aging traits

For survival time, we constructed Kaplan-Meier survival curve for all participants. After removing 14 cases with missing data, the survival function was computed for 4,490 samples with 1,282 deaths. A Cox proportional hazards regression with adjustment for age was performed to evaluate baseline predictors of survival time. Only factors that were significant from this analysis were included as covariates in the genetic association test. Survival analysis was done using the “survival” R package (Therneau and Grambsch, 2000).

For cognitive aging, we limited the analysis to 4,284 participants who had repeated measures of the 3MSE exam from baseline and at least 2 follow-up visits. For this subset, the retention rate is high and over 85% of the 4,284 participants have annual 3MSE scores for up to 6 years of follow-up from baseline. However, there is rapid decline in participant number in subsequent years. The follow-up rates for 11 visit years are provided in Supplementary Table S1. Original reports on the effect of HT on cognitive function in WHIMS were published in 2003 and 2004 (Rapp et al., 2003; Shumaker et al., 2003, 2004; Espeland et al., 2004). Here our primary interest is on the individual-level longitudinal trajectories in cognitive function and we used mixed effects modeling to estimate the rate of change in 3MSE score as a function of visit year with random intercept by subject and random slope by visit year (Rapp et al., 2003; Padula et al., 2016). This was also adjusted for baseline 3MSE score (R codes are provided in Supplementary Data S1). The rate-of-change coefficient as a function of visit year was extracted for each person and this was used as the quantitative trait for cognitive aging. We used age adjusted linear regression analysis to evaluate baseline predictors of this phenotype and only significant baseline factors were included as covariates in the genetic association test. Mixed effects modeling was done using “lme4” R package (Bates et al., 2015) and all statistical analyses were performed in R.

### Genetic data QC and analysis

The WHI Coordinating Center performed imputation against the 1,000 Genomes reference population (Genomes Project et al., 2010). The pre-imputation genotype quality control parameters are: sample call rate >97%, SNP call rate > 98%, Hardy Weinberg disequilibrium >1 × 10^−4^, and a minor allele frequency (MAF) cutoff of 1%. A panel of 5665 SNPs was used to compute an identity-by-descent (IBD) matrix using PLINK (Purcell et al., 2007). The Genetics and Epidemiology of Colorectal Cancer Consortium (GECCO) performed the IBD analysis for WHI (Peters et al., 2012). The GECCO group maintains a standard list of SNPs for genetic ancestry analysis that were selected according to recommendations by Lee et al. (2010). The panel of 5665 SNPs was in the intersection of that list and was used to perform IBD and population structure analyses for all the WHI datasets including WHIMS. Principal component (PC) analysis was then performed to derive the genetic population structure. Plot of the first two PCs shows that majority of the WHIMS participants, with the exception of a few individuals, group into one cluster (Supplementary Figure S1).

We focused on the 78 members of the *MRP* gene family and extracted 6959 imputed variants located within these genes. Gene coordinates were according to RefSeq annotations and based on GRCh37/Hg19 (Supplementary Table S2). Variants were further filtered using an imputation quality *R*^2^ ≥ 0.5 and MAF ≥ 0.01. The final list of 3,693 SNPs/variants was used in association test. Additionally, we extracted 6 SNPs in *APOE* with *R*^2^ ≥ 0.5 and MAF ≥ 0.01. The list of SNPs and associated quality scores and statistics are provided in Supplementary Data S1. The two SNPs, rs429358 and rs7412, define the *APOE-*ε*4* risk allele, and we dichotomized WHIMS samples to those with at least one dose of the risk allele, and those without (Goveas et al., 2016).

We applied an additive model and computed the dosage of the major allele from the imputed AA and AB genotype probabilities using the formula: allele dosage = 2 × prob(AA) + prob(AB) (Marchini and Howie, 2010). We used this imputed allelic dosage as the main predictor variable in the genetic association tests. To test the association with survival time, we applied two Cox proportional hazard models. Model 1 was adjusted for baseline age and the first three PCs of population structure. In model 2, we further adjusted for HT group assignment, *APOE-*ε*4* carrier status, and baseline variables that were significant predictors of survival time (3MSE score, income, depressed mood, smoking status, alcohol use, energy expenditure, and history of hypertension, CVD and cancer; regression models provided in Supplementary Data S1). After excluding 466 samples due to missing variables, the full Cox regression model was done on 4038 participants with 1136 deaths. We used a quantile-quantile (QQ) plot to compare between observed vs. expected *p*-values. For visualization, we generated Manhattan plots for the *MRP* SNPs using the R package “qqman” (Turner, 2014).

For cognitive aging, we used two linear regression models. Model 1 was adjusted for baseline age and PC1 to PC3 of population structure. Model 2 was further adjusted for HT group assignment, *APOE-*ε*4* carrier status, and baseline variables that were significantly associated with cognitive aging (baseline 3MSE score, income, and BMI; regression models provided in Supplementary Data S1). After excluding 395 samples due to missing data, the full regression model was done on a sample of 4,109 WHIMS participants. For an additive genetic model, if we have a marker with allele frequency of 0.15, the sample size of ~4,000 participants gives us approximately 75% power to detect a relatively high effect of 0.30 at an uncorrected *p*-value of 0.00001 (power calculation was done using the R package GeneticsDesign version 1.44.0).

### Gene-set analyses for *MRP* family

We applied two distinct pathway level (i.e., gene-set) methods to calculate the combined effect of *MRPs*. GATES applies an extended Simes procedure based on a gene and pathway level minimum *p*-value method (Gui et al., 2011; Li et al., 2011). The second method, MAGMA, combines SNP level statistics within a gene and applies a regression procedure to calculate the combined gene-set statistics (de Leeuw et al., 2015, 2016). For MAGMA, we used the top Chi-square option with adaptive permutation (minimum of 1,000 permutations) to generate gene level *p*-values. Both GATES and MAGMA incorporate the linkage disequilibrium (LD) structure and effectively correct for correlation between SNPs and gene size bias. For LD calculation, genotypes for the 4504 WHIMS participants were hard-called from imputed dosages and loaded to PLINK to generate a 3,693 × 3,693 LD matrix (Purcell et al., 2007). GATES was done using the R “aSPU” package (Pan et al., 2014), and the MAGMA was done using the application version v1.04.

## Results

### Baseline predictors of aging traits

During enrollment to WHIMS, participants were over 65 years old and free of dementia. Demographic and health characteristics of participants at baseline are provided in Table 1. Twenty-five percent were carriers of at least one dose of the *APOE-*ε*4* risk allele.

**Table 1:** Demographic characteristics and health and cognitive profiles at baseline.

**Variables^a^**	**WHIMS^b^ (*N* = 4,504)**
Age (years)	70.02 (3.78)
Depressed mood (CES-D/DIS)^c^	0.03 (0.1)
Recreational energy expenditure (MET-hours/week)^d^	11.67 (13.28)
Body mass index (kg/m^2^)	28.24 (5.52)
Global cognitive score (3MSE)^e^	95.92 (3.68)
**INCOME**	
<$19,999	946 (22%)
$20K to $34,999	1,374 (32%)
$35K to $49,999	920 (22%)
$50K to $75,999	642 (15%)
>$75K	365 (9%)
Don't know or missing	257
**EDUCATION**	
<high school	237 (5%)
High school or GED	997 (22%)
Vocation or some college	1,812 (40%)
College graduate	401 (9%)
Post-graduate or professional	1,044 (23%)
Don't know or missing	13
**HORMONE THERAPY ARM**	
Estrogen-alone intervention	712 (16%)
Estrogen+Progesterone intervention	1,501 (33%)
Estrogen-alone control	747 (17%)
Estrogen+Progesterone control	1,544 (34%)
**SMOKING STATUS**	
Never Smoked	2,344 (52%)
Past Smoker	1,808 (41%)
Current Smoker	287 (6%)
Missing	65
**ALCOHOL USE**	
Non drinker	506 (11%)
Past drinker	760 (17%)
<1 drink per month	551 (12%)
<1 drink per week	877 (20%)
1 to <7 drinks per week	1,159 (26%)
7+ drinks per week	617 (14%)
Missing	34
**HYPERTENSION EVER**	
No	2,856 (64%)
Yes	1,607 (36%)
Missing	41
**HIGH CHOLESTEROL EVER**	
No	3,658 (82%)
Yes	791 (18%)
Missing	55
**CARDIOVASCULAR DISEASE EVER**	
No	3,742 (84%)
Yes	707 (16%)
Missing	55
**CANCER EVER**	
No	4,327 (97%)
Yes	150 (3%)
Missing	27
***APOE-ε4* CARRIER**	
No	3,370 (75%)
Yes	1,134 (25%)

a *Mean (SD) for continuous variables and N (percent) for non-numeric variables*.

b *WHIMS, Women's Health Initiative Memory Study*.

c *Center for Epidemiological Studies; depression scale (CES-D, short form)*.

d *Metabolic Equivalent of Task*.

e *Modified Mini-Mental State Examination*.

**Figure 1:**
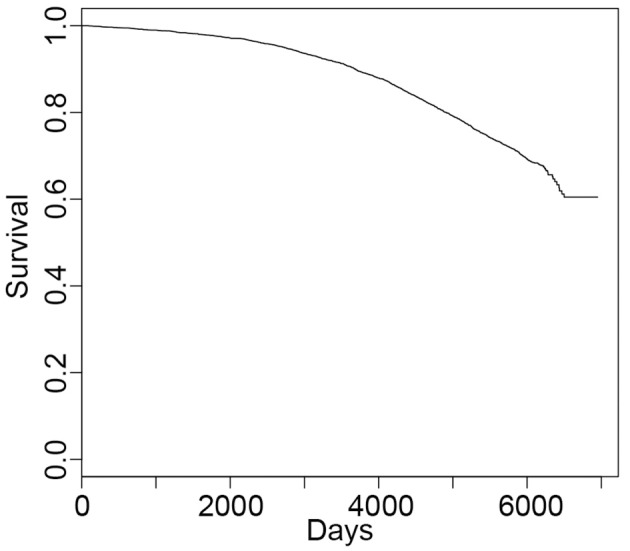
Survival curve for all-cause mortality. The median time to death is 5,802 days from enrollment (average ± *SD* = 5,251 ± 1,207 days) with 1282 deaths (*N* = 4,504 and 14 with missing data).

For survival, the median days from enrollment to end of follow-up or all discovered death is 5,802 (Figure 1). As expected, older age at baseline is associated with a higher risk of death (hazard ratio HR = 1.12; 95% confidence interval CI = 1.11, 1.14; Supplementary Table S3). After controlling for age, other significant risk factors were depressed mood, smoking, alcohol use, and history of hypertension, CVD and cancer, and being a carrier of the *APOE-*ε*4* allele (Supplementary Tables S3, S4). In contrast, higher recreational energy expenditure, and higher baseline 3MSE score and income were associated with reduced risk.

**Figure 2:**
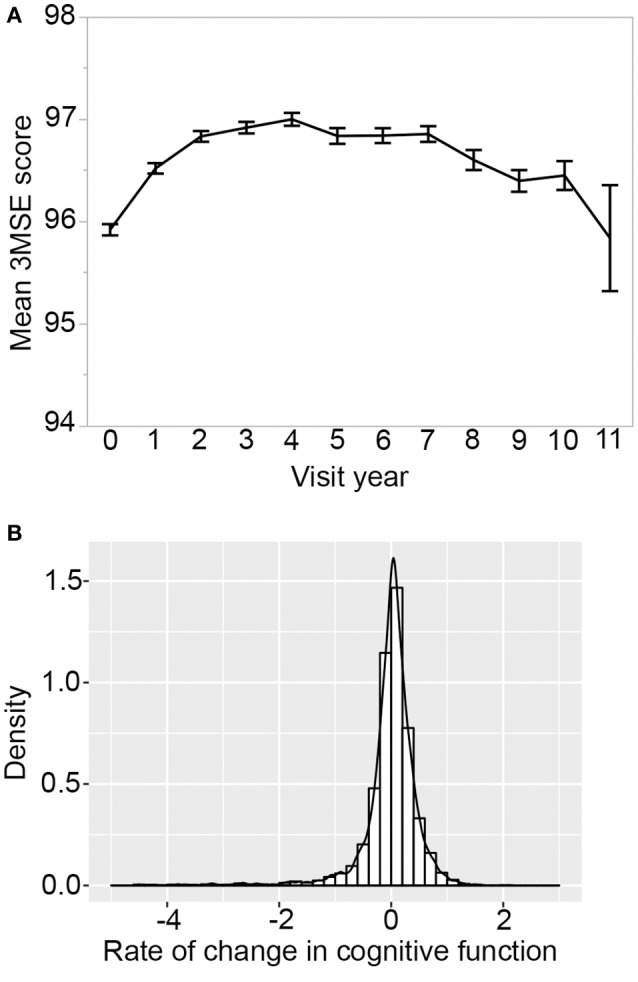
Cognitive change over time. **(A)** Longitudinal plot of average 3MSE scores from baseline and up to 11 years of annual follow-up visits. On average, there are *N* = 3,166 participants per year, and this decreased to only 239 participants by year 11. Error bar is standard error. **(B)** Distribution of rate-of-change shows significant variation in cognitive aging trajectories among participants. Four thousand two hundred eighty-four WHIMS participants with 3MSE scores available at baseline and at least two follow-up visit years were used to derive regression slope over time.

For cognitive aging, the longitudinal plot of the average 3MSE scores shows marked change over the course of study. As reported previously (Rapp et al., 2003; Espeland et al., 2004), there is an increase over the first four years due to positive practice effect, followed by a decline in subsequent years (Figure 2A). On average, participants have 8 repeated measures of cognitive function. The number of participants and average scores stratified by HT groups are provided in Supplementary Table S5. 4,284 participants have valid 3MSE scores from baseline and at least two follow-up years and we considered this set to determine person-specific longitudinal trajectories in cognitive function. The rate of change in cognitive function varies widely among the participants (Figure 2B). Age at baseline was the strongest predictor with higher age associated with greater rate of decline (Supplementary Table S3). After controlling for age, higher baseline 3MSE score, lower income, and being carriers of the *APOE-*ε*4* allele were associated with a significantly higher rate of decline. Higher baseline BMI, on the other hand, is associated with lower rate of decline (Supplementary Tables S3, S4). While overall decline rate is higher in the hormone intervention groups relative to placebo, this did not reach statistical significance [*F*_(2, 4, 280)_ = 1.87, *p*-value = 0.15].

### SNP-level analysis between the *MRPs* and aging traits

**Figure 3:**
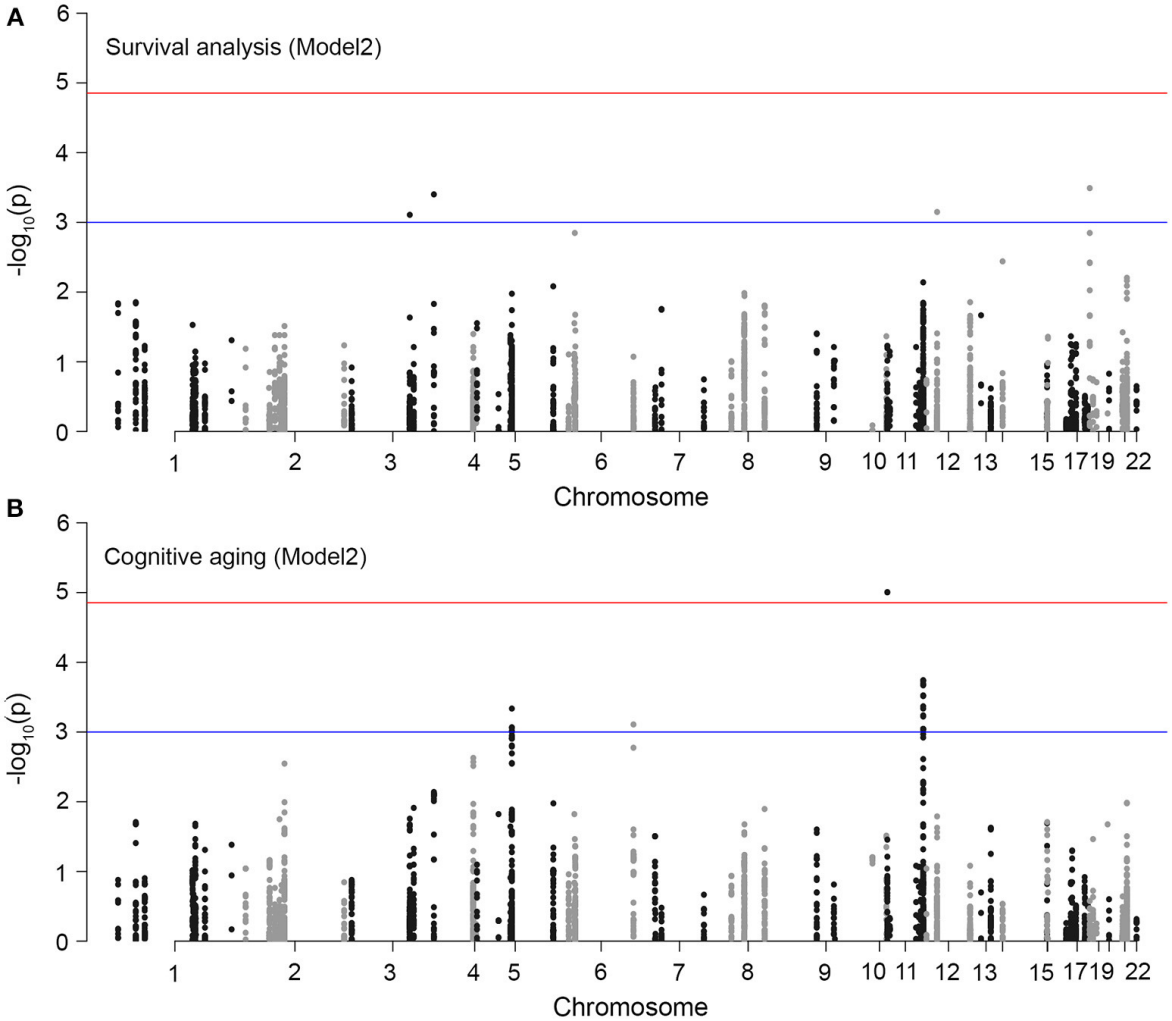
Association plots for aging traits in WHIMS. Each point in the plots represents a variant in an *MRP* gene (total number of markers = 3,693). The chromosomal position is on the x-axis and the y-axis shows the −log_10_(*p*-value) of association with **(A)** survival time, and **(B)** cognitive aging. The top horizontal red line denotes the multiple-test corrected significant *p*-value threshold (1.3 × 10^−5^). We set a suggestive threshold of 1 × 10^−3^ (denoted by bottom blue line).

**Figure 4:**
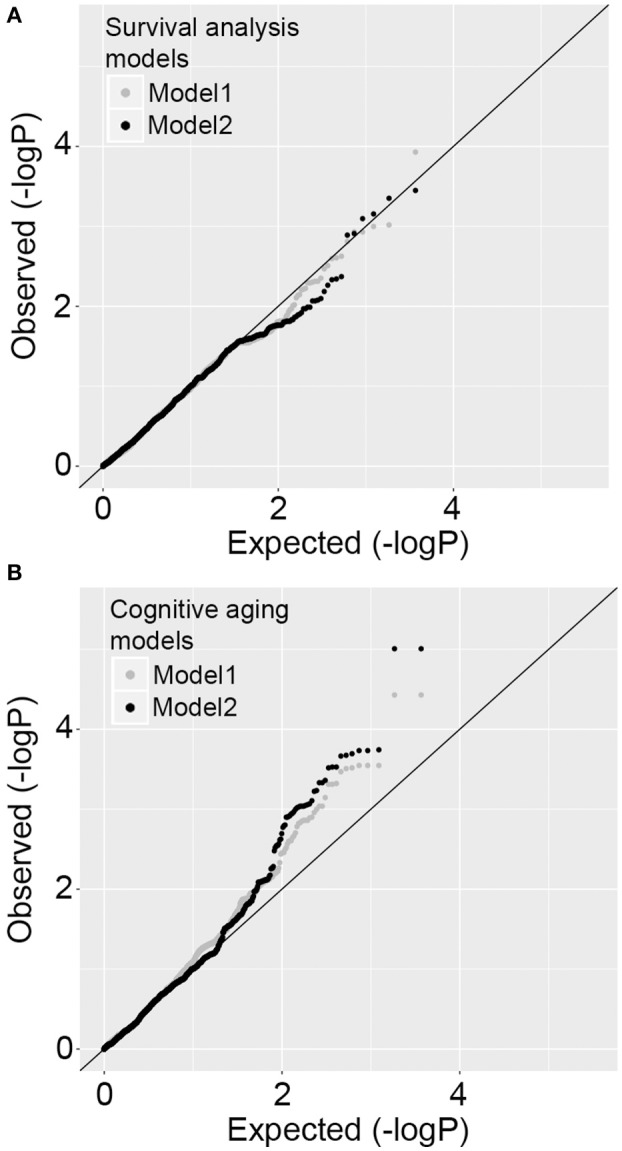
Quantile-Quantile plots for SNP level association tests. **(A)** *P*-value distributions for survival time measured as time to all-cause mortality using Cox regression model 1 and model 2. There is no deviation from expected distribution. **(B)** *P*-value distributions for cognitive aging measured as the rate of change in cognitive function using regression model 1 and model 2. There is significant deviation from expected distribution.

We first tested SNP level association between the 3,693 SNPs/variants and survival time. Model 1 was adjusted for baseline age and population structure, and model 2 was additionally adjusted for *APOE*-ε4 status, HT group assignment, and baseline variables significantly associated with survival. No variant reached statistical significance at the Bonferroni corrected threshold of alpha 0.05 (0.05/3693 = 0.00001) for either models. The location of the SNPs and association *p*-values are shown in Figure 3A. The QQ plots indicate no significant association with survival time (Figure 4A; full results in Supplementary Data S1). The *APOE-*ε*4* SNPs—rs429358 and rs7412—show weak association with survival (*p*-value = 0.04 and *p*-value = 0.02, respectively; Table 2).

**Table 2:** *APOE* SNPs ranked by association with aging traits.

**SNP**	**Major/Minor**	**Survival time^a^**		**Cognitive aging^b^**	
		**HR**	***P*-value**	**Beta**	***P*-value**
*rs429358*^c^	T/C	0.88	0.04	0.21	4.9E–28
*rs769449*	G/A	0.84	0.01	0.21	2.0E–23
*rs440446*	C/G	0.96	0.42	−0.05	0.0009
*rs769450*	G/A	1.05	0.30	−0.04	0.003
*rs7412*^c^	C/T	1.21	0.02	−0.06	0.02
*rs769448*	C/T	0.84	0.26	−0.05	0.36

a *Cox regression with adjustment for population structure, hormone therapy arm and significant baseline covariates of survival time; HR is hazards ratio per copy of major allele*.

b *Linear regression with adjustment for population structure, hormone therapy arm and significant baseline covariates of cognitive change; beta is the linear regression coefficient (i.e., cognitive change per copy of major allele)*.

c *APOE-ε4 SNPs; minor allele C in rs429358 and major allele C in rs7412 are associated with increased risk*.

We used a similar model 1 and model 2 approach for cognitive aging. Two SNPs in LD in *MRPL23* (rs189661478, rs187127498) had a significant association at *p*-value = 9 × 10^−6^ (Figure 3B). These two SNPs are located in the last intron of *MRPL23* with MAF = 0.01 and high imputation quality (*R*^2^ ≥ 0.78). The QQ plots also indicate significant deviation of observed *p*-values from the expected null distribution (Figure 4B; full results in Supplementary Data S1). Of the 78 *MRP* genes, 33 have at least one SNP with nominal *p*-value ≤ 0.05 (Table 3).

**Table 3:** *MRP* genes ranked by minimum *p*-values and gene level association with cognitive aging.

**MRP**	**Chr**	**Minimum P in each** ***MRP***					**Gene-wise P^c^**	
		**SNP^a^**	**Major/Minor**	**Beta^a^**	**minP^a^**	**nSNP^b^**	**GATES**	**MAGMA**
*MRPL23*	11	*rs189661478*	G/C	0.39	9.9E–06	41	0.0002	0.0003
*MRPL48*	11	*rs149591437*	T/−	0.12	0.0002	319	0.008	0.01
*MRPL18*	6	*rs111461592*	G/A	0.16	0.0008	52	0.01	0.01
*MRPS27*	5	*rs147356411*	C/T	0.42	0.0005	202	0.02	0.04
*MRPS12*	19	*rs730078*	A/G	0.03	0.02	1	0.02	0.03
*MRPS30*	5	*rs35850760*	A/G	−0.10	0.02	9	0.05	0.06
*MRPL47*	3	*rs2339844*	A/C	0.07	0.007	33	0.06	0.07
*MRPL55*	1	*rs849749*	C/T	0.03	0.04	3	0.12	0.11
*MRPL1*	4	*rs112015199*	C/T	−0.11	0.002	262	0.13	0.15
*MRPS10*	6	*rs138935030*	G/A	0.10	0.02	53	0.13	0.13
*MRPL46*	15	*rs17188013*	A/G	−0.05	0.02	7	0.13	0.12
*MRPS9*	2	*rs147361163*	A/G	0.31	0.003	289	0.15	0.15
*MRPS22*	3	*rs112795230*	G/−	−0.05	0.01	48	0.20	0.24
*MRPL28*	16	*rs35604057*	C/T	−0.04	0.02	22	0.20	0.19
*MRPS36*	5	*rs181429701*	C/T	0.29	0.02	37	0.21	0.22
*MRPL2*	6	*rs1214704*	G/A	−0.14	0.03	17	0.24	0.21
*MRPS15*	1	*rs16823042*	T/C	−0.04	0.02	43	0.25	0.26
*MRPL22*	5	*rs72799532*	T/C	0.25	0.01	73	0.26	0.25
*MRPL13*	8	*rs117818575*	A/G	0.26	0.01	79	0.26	0.22
*MRPL3*	3	*rs142756323*	C/A	0.20	0.02	102	0.27	0.39
*MRPL43*	10	*rs11591349*	A/T	0.03	0.03	23	0.27	0.34
*MRPL32*	7	*rs598810*	G/A	0.05	0.03	24	0.27	0.25
*MRPL4*	19	*rs2304236*	A/C	−0.11	0.03	17	0.29	0.26
*MRPS5*	2	*rs201027877*	C/−	0.11	0.02	31	0.31	0.30
*MRPL50*	9	*rs13284088*	T/A	0.07	0.03	28	0.33	0.32
*MRPS18A*	6	*rs76134013*	C/T	0.06	0.04	49	0.36	0.51
*DAP3*	1	*rs9426933*	T/C	−0.03	0.02	64	0.40	0.34
*MRPL27*	17	*rs4793651*	A/G	0.03	0.05	21	0.40	0.38
*MRPS28*	8	*rs13274346*	C/T	−0.09	0.02	203	0.48	0.61
*MRPS31*	13	*rs9577129*	A/G	−0.05	0.02	96	0.50	0.39
*MRPS35*	12	*rs146117897*	−/TTG	0.07	0.02	119	0.54	0.52
*MRPS6*	21	*rs62212081*	G/T	0.19	0.01	148	0.57	0.48
*MRPS14*	1	rs528474110	T/−	−0.11	0.05	27	0.62	0.48

a *Variant with lowest p-value (minP) in each gene; beta is linear regression coefficient, i.e., cognitive change per copy of major allele with adjustment for population structure, APOE- ε4 status, hormone therapy arm and significant baseline covariates of cognitive aging (regression model 2)*.

b *Number of SNPs tested within a gene*.

c *Gene level p-value computed by GATES and MAGMA (after a minimum of 1,000 permutations)*.

The *APOE* SNP, rs429358, has the strongest association with cognitive aging (*p*-value = 5 × 10^−28^) (Supplementary Data S1; Table 2). SNP rs7412 also has a nominal association (*p*-value = 0.02). Another SNP in *APOE*, rs769449, is also significantly associated with this trait (*p*-value = 2 × 10^−23^), but this drops to 0.003 when controlled for the *APOE-*ε*4* risk status indicating that this is not entirely independent of the *APOE-*ε*4* SNPs.

### Gene-set test for the *MRP* family and cognitive aging

We then tested if the *MRP* family as a group is associated with cognitive aging. Gene-wise *p*-values were derived using two methods: GATES and MAGMA. After adjustment for gene-wise multiple test and LD structure, *MRPL23* has a significant gene level *p*-value of 0.0002 (GATES) and 0.0003 (MAGMA) (Bonferonni threshold is 0.05/78 = 0.0006) (Table 3). Out of the 78, only 6 *MRPs* have gene-wise *p*-value ≤ 0.05. Following the gene level tests, we performed pathway level gene-set analyses. Only the GATES procedure detected modest but significant association between *MRP* family and cognitive aging at *p*-value = 0.01. The gene-set test was not significant with the MAGMA procedure.

## Discussion

### The *MRP* gene family and aging traits

Our main motivation to test the *MRPs* comes from studies carried out in model organisms showing that mutations in members of this gene family contribute to aging and longevity. The influence of the *MRPs* on lifespan is conserved across phyla, and decreased expression of *Mrps*, either due to natural genetic variation or as a result of genetic manipulation, has a life-extending effect in both mice and *C. elegans* (Houtkooper et al., 2013; Mouchiroud et al., 2013). This effect has been attributed to perturbations in the balance between nuclear and mitochondria encoded proteins. The stoichiometric imbalance, termed as mitonuclear imbalance, triggers the mitochondrial unfolded protein response (UPR^mt^), which is considered to be a conserved longevity pathway (Wang and Hekimi, 2015). The goal of this study was to test if we can find evidence for this effect in humans using the phenotypic and genotypic data from the WHIMS cohort. In essence, this work is a focused genetic study in which we leveraged epidemiological and genetic data from humans to test if there is detectable genetic association between the *MRP* gene family and complex aging traits.

For the gross outcome, i.e., all-cause mortality, the *p*-value distribution for the *MRP* variants did not deviate from the null hypothesis. The lack of association is not entirely surprising given the relatively small sample size of our study, and the complex and inherently heterogeneous nature of the phenotype (i.e., different reasons including aging independent factors can lead to mortality). In addition to genetics, numerous lifestyle and demographic variables also influence lifespan. In the WHIMS cohort, we find that higher physical activity (measured by recreational energy expenditure), and higher cognitive function and income at baseline are associated with longer survival time. In contrast, smoking, alcohol use, depressed mood and health conditions (history of hypertension, CVD, cancer) at baseline are associated with mortality risk. Our results are consistent with previous studies in the larger WHI cohort that have shown that higher physical activity at baseline and psychosocial wellbeing can predict survival time and health at advanced age (Woods et al., 2012; Seguin et al., 2014). The phenotypic complexity and genetic heterogeneity may also partly explain why genes linked to lifespan in animal models show no genome-wide significant association in humans (Walter et al., 2011). Additionally, we focused specifically on the family of 78 genes and did not consider other downstream mediators such as members of the UPR^mt^ pathway. While the negative finding in this study does not discount the involvement of mitonuclear imbalance response in human health and aging, our results show that the link between *MRP* gene variants and lifespan is not replicated in humans.

Following the analysis of gross survival time, we used cognitive change as a more specific indicator of age-related functional decline, particularly brain aging. Cognitive performance is a strong predictor of health during aging and overall longevity (Riley et al., 2005; Terracciano et al., 2008; Batty et al., 2009). In this regard, genes related to mitochondrial function are prime candidate mediators of the crosstalk between brain function and overall aging and this organelle has been implicated in both lifespan regulation and the development of dementia and Alzheimer's disease (Bishop et al., 2010; Swerdlow, 2011; Garcia-Escudero et al., 2013; Gkikas et al., 2014; Picard and McEwen, 2014). The SNP and gene level results support an association between the *MRPs* and cognitive aging. In particular, we find significant SNP level and gene level signal for *MRPL23*. The two SNPs (rs189661478 and rs187127498) that show significant association with cognitive aging are both located in the last intron of *MRPL23* and are in strong LD. No other neighboring SNPs in our data are in LD with the pair. Notably for rs189661478, SNP annotations in the Ensemble browser (http://grch37.ensembl.org) indicate that it may be a splice variant and may have functional consequence.

WHIMS was designed to evaluate the impact of HT on cognitive function. Previous studies in WHIMS found that HT resulted in increased risk for cognitive decline (Rapp et al., 2003; Shumaker et al., 2003, 2004; Espeland et al., 2004, 2010; Goveas et al., 2011, 2014; Haring et al., 2013; Vaughan et al., 2013). A more recent study by Goveas et al. (2016) examined predictors of cognitive function in an older subset of WHIMS (i.e., over 80 years). The work by Goveas relied on categorical classification into normal and adjudicated cases of cognitive impairment, and they found no sustained effect of HT, at least in this aged cohort. In the present work, while the decline rate in cognitive function is higher in the two HT groups relative to control groups, this difference does not reach statistical significance. As in Goveas et al. (2016), we find that lower income at baseline is associated with cognitive decline. Somewhat unexpected, we find that higher 3MSE score at baseline is associated with a greater rate of decline, whereas higher baseline BMI is slightly protective. In this subset of WHIMS, some of the women who scored low at baseline (3MSE < 80) either remained stable or made gains in follow-up years, whereas those who scored high at baseline showed, on average, a more negative longitudinal trajectory over the course of the study. In terms of BMI, epidemiological studies generally associate higher BMI and obesity with increased risk for cognitive impairment and Alzheimer's disease (Yaffe et al., 2009; Barnes and Yaffe, 2011). However, a recent meta-analysis found a more complex association with higher BMI at mid-life increasing risk and higher BMI at late life decreasing risk (Xu et al., 2015). Adjusting for these covariates in the genetic association test increased the strength of association between the *MRP* SNPs and cognitive aging.

Similar to work on the genetics of age-related cognitive decline (de Jager et al., 2012; Sherva et al., 2014; Zhang and Pierce, 2014), we used the individual-level slope of change to capture cognitive aging. Consistent with Goveas et al. (2016), we also find that the *APOE-*ε*4* allele is strongly associated with cognitive aging. The *APOE-*ε*4* allele also shows a nominally significant association with lifespan. The *APOE* locus presents a prime example of the pleiotropic influence of genes on both cognitive function and lifespan and has been consistently implicated in Alzheimer's disease and human longevity (Corder et al., 1993). Recently, Davies et al. (2014) showed that the *APOE* SNP, rs429358, is associated with non-pathological aging with a more prominent effect in women. We replicate this strong effect in WHIMS.

### Pathway-level analysis for the *MRP* gene family

The standard SNP level test treats a single variant as an independent functional unit and fails to capture the summarized effect of multiple variants. Additionally, SNP level associations may have poor replication if the polymorphism is specific to a particular population. For instance, the two significant *MRPL23* SNPs, rs189661478 and rs187127498, have minor allele frequencies of 0.01 in European populations. However, the minor allele is not found in non-European reference panels in the 1,000 Genomes Phase 3 populations. The SNP level association is therefore specific to European ancestral groups. In contrast, gene level associations may be more robust to population specific differences in SNP frequencies since it treats the gene as a functional unit, and summarizes the association signal arising from multiple variants within the gene. And when it comes to gene families such as the *MRPs*, it may be more meaningful to consider the group as a set rather than individual SNPs. For instance, in the work done in mice and *C. elegans* (Houtkooper et al., 2013), lifespan was correlated with the expression of not just one but several members of the *Mrp* family. The *MRPs* in humans constitute a relatively large gene family with 78 canonical members and the variants distributed among these genes may exert a combined influence on a phenotype.

A number of different strategies have been developed to test pathway level association. These methods, in essence, treat the gene or gene-set as the functional unit. The underlying statistics can, however, vary greatly from method to method (de Leeuw et al., 2016; Kwak and Pan, 2016). We implemented two very distinct procedures, GATES and MAGMA, to test if the signal for the *MRP*s is robust to both algorithms. GATES applies a minimum *p*-value selection and is better powered to detect the effect of a few but strong associations. MAGMA, on the other hand, combines all the gene level *p*-values to derive the pathway statistic, and it more effective if many genes contribute to the gene level association signal. We found modest but significant association with GATES but not with MAGMA. This is likely because while the QQ-plot for the MRP SNPs shows a strong deviation from the null hypothesis, when corrected for gene-wise multiple tests and LD structure, only 5 *MRP* genes have *p*-values < 0.05.

### Limitations

An important limitation is that WHIMS is by no means a representative population. It was specifically designed to study the effect of HT on cognitive function and dementia risk in post-menopausal women. This study benefits from the detailed longitudinal cognitive assessment and demographic and health data. However, any sex-specific effect cannot be accounted for. Additionally, the participants in this study are Caucasians. This limits the generalizability of the association between the *MRPs* and cognitive aging.

An important next step is to replicate and verify the association of the *MRPs* on cognitive aging in other longitudinal cohorts. Pertinent to this is the genome-wide study of nonpathological cognitive aging by Davies et al. (2014). Here they reported a suggestive gene-based association (*p*-value < 0.01) for *MRPS28* specifically in the female subset. Another work is the GWAS by Sherva et al. (2014) that also used a similar quantitative measure of cognitive decline in patients with Alzheimer's disease. Their study found a strong association between a SNP in *MRPL10* (rs62076130) and rate of cognitive decline in Alzheimer's cases (44% females; *p*-value = 7.8 × 10^−7^). We did not replicate these gene-level (for *MRPS28*) and SNP-level (for rs62076130) associations in WHIMS. However, the collective evidence does implicate the *MRP* gene family in human cognitive aging. It will be important that the follow-up replication study for this gene-set association include cohorts that have both male and female participants, and are more diverse in terms of ethnicity and genetic ancestry.

To conclude, we provide evidence that variants in *MRPs* may influence cognitive aging in older women. However, we did not detect an association with overall lifespan.

## Author contributions

KM: study design, data analysis and interpretation, initial manuscript preparation, and final manuscript approval. BS and SR: helped with data analysis and interpretation, provided access to WHIMS data, and approval of final manuscript. RWW: study design, contributed to manuscript, and final manuscript approval. KJ and RBW: provided oversight and guidance with WHI, contributed to manuscript, and final manuscript approval.

### Conflict of interest statement

The authors declare that the research was conducted in the absence of any commercial or financial relationships that could be construed as a potential conflict of interest.
