# Supplementary material for: Genetic Analysis of Mitochondrial Ribosomal Proteins and Cognitive Aging in Postmenopausal Women
Source: Front Genet. 2017 Sep 21;8:127. doi: 10.3389/fgene.2017.00127 (PMC5613226; doi:10.3389/fgene.2017.00127)
Supplement: Supplementary file 1 [file Table1.docx]

**Supplementary Figure**

**
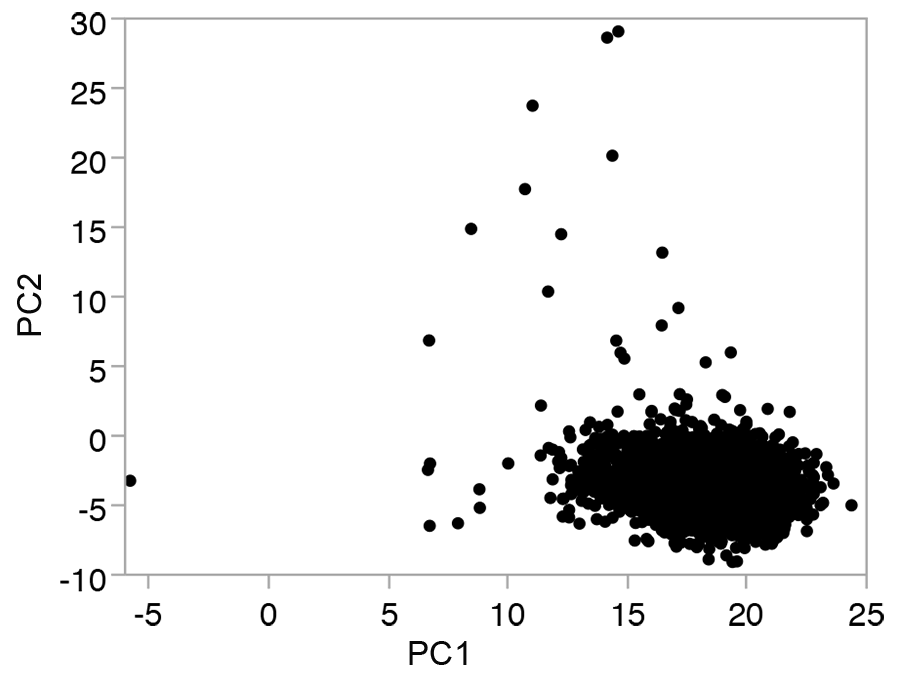
**

**Figure S1.** Principal component analysis (PCA) of population structure in WHIMS (N = 4504). The PCA plot for the first two principal components (PC1 and PC2) shows that, with the exception of few outliers, majority of the women group into one cluster.

**Supplementary tables**

**Supplementary Table S1:** Follow-up rate for annual Modified Mini-Mental State Exam in the WHIMS subset used for genetic association test

| **Total N** | **N** | **Follow-up rate (%)** |
| --- | --- | --- |
| Baseline | 4284 |  |
| Visit year 1 | 4205 | 98 |
| Visit year 2 | 4130 | 96 |
| Visit year 3 | 4116 | 96 |
| Visit year 4 | 3931 | 92 |
| Visit year 5 | 3792 | 89 |
| Visit year 6 | 3703 | 86 |
| Visit year 7 | 3156 | 74 |
| Visit year 8 | 2436 | 57 |
| Visit year 9 | 2250 | 53 |
| Visit year 10 | 1471 | 34 |
| Visit year 11 | 239 | 6 |

**Supplementary Table S2:** List of *MRP* genes and genomic coordinates

| MRP | Position (GRCh37/Hg19) |
| --- | --- |
| *MRPL20* | chr1:1337275–1342693 |
| *MRPS15* | chr1:36921361–36930040 |
| *MRPL37* | chr1:54665839–54684056 |
| *MRPS21* | chr1:150266261–150281414 |
| *MRPL9* | chr1:151732118–151736392 |
| *DAP3* | chr1:155658881–155708800 |
| *MRPL24* | chr1:156707093–156710923 |
| *MRPS14* | chr1:174982093–174992591 |
| *MRPL55* | chr1:228294379–228297013 |
| *MRPL33* | chr2:27994583–28002608 |
| *MRPL53* | chr2:74699084–74699942 |
| *MRPL19* | chr2:75873908–75889334 |
| *MRPL35* | chr2:86426555–86440477 |
| *MRPS5* | chr2:95752951–95787754 |
| *MRPL30* | chr2:99797541–99816020 |
| *MRPS9* | chr2:105654482–105716418 |
| *MRPL44* | chr2:224822120–224832431 |
| *MRPS25* | chr3:15090018–15106816 |
| *MRPL3* | chr3:131181044–131221860 |
| *MRPS22* | chr3:139062860–139075887 |
| *MRPL47* | chr3:179306254–179322434 |
| *MRPL1* | chr4:78783804–78873944 |
| *MRPS18C* | chr4:84377084–84382876 |
| *MRPL36* | chr5:1798498–1799956 |
| *MRPS30* | chr5:44809026–44815618 |
| *MRPS36* | chr5:68513572–68525985 |
| *MRPS27* | chr5:71515235–71616084 |
| *MRPL22* | chr5:154320632–154348971 |
| *MRPS18B* | chr6:30585485–30594174 |
| *MRPS10* | chr6:42174538–42185633 |
| *MRPL2* | chr6:43021766–43027283 |
| *MRPS18A* | chr6:43638933–43655549 |
| *MRPL14* | chr6:44081372–44095191 |
| *MRPL18* | chr6:160211491–160219461 |
| *MRPL32* | chr7:42971938–42977453 |
| *MRPS24* | chr7:43906156–43909145 |
| *MRPS17* | chr7:56019610–56023033 |
| *MRPS33* | chr7:140705960–140714781 |
| *MRPL15* | chr8:55047780–55061074 |
| *MRPS28* | chr8:80831094–80942506 |
| *MRPL13* | chr8:121408082–121457647 |
| *MRPL50* | chr9:104152248–104160919 |
| *MRPS2* | chr9:138392476–138396519 |
| *MRPL41* | chr9:140446308–140447007 |
| *MRPS16* | chr10:75008600–75012451 |
| *MRPL43* | chr10:102737578–102747272 |
| *MRPL23* | chr11:1968501–1977839 |
| *MRPL17* | chr11:6701615–6704632 |
| *MRPL16* | chr11:59573607–59578345 |
| *MRPL49* | chr11:64889654–64894841 |
| *MRPL11* | chr11:66202545–66206319 |
| *MRPL21* | chr11:68658745–68671303 |
| *MRPL48* | chr11:73498916–73575656 |
| *MRPL51* | chr12:6601315–6602471 |
| *MRPS35* | chr12:27863705–27909237 |
| *MRPL42* | chr12:93861265–93897548 |
| *MRPL57* | chr13:21750371–21753220 |
| *MRPS31* | chr13:41303431–41345347 |
| *MRPL52* | chr14:23299091–23304246 |
| *MRPL46* | chr15:89002708–89010633 |
| *MRPS11* | chr15:89010683–89021861 |
| *MRPL28* | chr16:417383–420569 |
| *MRPS34* | chr16:1821895–1823140 |
| *MRPL45* | chr17:36452988–36479101 |
| *MRPL10* | chr17:45900637–45908907 |
| *MRPL27* | chr17:48445227–48450562 |
| *MRPS23* | chr17:55916286–55927433 |
| *MRPS7* | chr17:73257748–73262457 |
| *MRPL38* | chr17:73894723–73901181 |
| *MRPL12* | chr17:79670399–79674556 |
| *MRPL54* | chr19:3762664–3767563 |
| *MRPL4* | chr19:10362639–10370736 |
| *MRPL34* | chr19:17416476–17417652 |
| *MRPS12* | chr19:39421347–39423659 |
| *MRPS26* | chr20:3026674–3028896 |
| *MRPL39* | chr21:26957967–26979801 |
| *MRPS6* | chr21:35445822–35515334 |
| *MRPL40* | chr22:19420035–19423596 |

**Supplementary Table S3. Baseline predictors of aging traits (continuous variables)**

|  | **Survival time^a^** | | **Cognitive aging^b^** | |
| --- | --- | --- | --- | --- |
| **Baseline variables** | **HR (95% CI)** | **P-value** | **Beta (SE)** | **P-value** |
| Age (years) | 1.12 (1.11, 1.14) | <2E-16 | -0.03 (0.003) | <2E-16 |
| Depressed mood (Burnam scale) | 1.74 (1.06, 2.86) | 0.03 | -0.018 (0.096) | 0.85 |
| Recreational energy expenditure (MET-hours/week) | 0.99 (0.99, 1) | 0.002 | -0.001 (0.001) | 0.21 |
| Body mass index (kg/m2) | 1.01 (1, 1.02) | 0.08 | 0.004 (0.002) | 0.03 |
| Global cognitive score (3MSE) | 0.95 (0.94, 0.97) | 2.6E-12 | -0.022 (0.003) | 2.0E-15 |

^a^Cox regression with adjustment for baseline age; HR is hazards ratio and CI is confidence interval

^b^Linear regression with adjustment for baseline age; Beta is coefficient in linear regression model; only samples with at least three valid 3MSE scores were used for longitudinal analysis of cognitive aging.

**Supplementary Table S4. Supplemental Table 1. Baseline predictors of aging traits (categorical variables)**

|  | **Survival time^a^** | | **Cognitive aging^b^** | |
| --- | --- | --- | --- | --- |
| **Variables** | **Mean follow-up in days**  **(#deaths/total)** | **P-value** | **Mean cognitive decline**  **(SD)** | **P-value** |
| Income |  |  |  |  |
| <$19,999 | 5058 (331/946) | 2.1E-06 | -0.095 (0.709) | 0.001 |
| $20K to $34,999 | 5221 (412/1374) |  | -0.029 (0.672) |  |
| $35K to $49,999 | 5354 (231/920) |  | 0.001 (0.532) |  |
| $50K to $75,999 | 5314 (157/642) |  | -0.009 (0.612) |  |
| >$75K | 5454 (81/365) |  | -0.012 (0.634) |  |
| Don't know | 5299 (22/94) |  | -0.059 (0.661) |  |
| Missing | 5312 (48/163) |  | -0.070 (0.946) |  |
| Education |  |  |  |  |
| < high school | 4941 (77/237) | 0.28 | -0.064 (0.803) | 0.34 |
| High school or GED | 5255 (273/997) |  | -0.008 (0.651) |  |
| Vocation or some college | 5258 (530/1812) |  | -0.03 (0.662) |  |
| College graduate | 5367 (108/401) |  | -0.043 (0.476) |  |
| Post-graduate or professional | 5267 (289/1044) |  | -0.054 (0.665) |  |
| Missing | 4800 (5/13) |  | -0.332 (1.074) |  |
| Hormone therapy arm |  |  |  |  |
| Estrogen-alone intervention | 5263 (197/712) | 0.75 | -0.033 (0.731) | 0.15 |
| Estrogen+Progesterone intervention | 5256 (433/1501) |  | -0.058 (0.658) |  |
| Estrogen-alone control | 5235 (218/747) |  | -0.003 (0.571) |  |
| Estrogen+Progesterone control | 5249 (434/1544) |  | -0.026 (0.654) |  |
| Smoking status |  |  |  |  |
| Never Smoked | 5358 (579/2344) | <2E-16 | -0.035 (0.671) | 0.59 |
| Past Smoker | 5226 (535/1808) |  | -0.027 (0.635) |  |
| Current Smoker | 4663 (138/287) |  | -0.038 (0.595) |  |
| Missing | 4702 (30/65) |  | -0.197 (0.856) |  |
| Alcohol use |  |  |  |  |
| Non drinker | 5240 (136/506) | 0.01 | -0.102 (0.805) | 0.19 |
| Past drinker | 5122 (240/760) |  | -0.02 (0.663) |  |
| <1 drink per month | 5257 (160/551) |  | -0.043 (0.655) |  |
| <1 drink per week | 5258 (252/877) |  | -0.028 (0.523) |  |
| 1 to <7 drinks per week | 5340 (291/1159) |  | -0.032 (0.749) |  |
| 7+ drinks per week | 5243 (193/617) |  | 0.007 (0.457) |  |
| Missing | 5123 (10/34) |  | -0.205 (0.579) |  |
| Hypertension ever |  |  |  |  |
| No | 5318 (720/2856) | 3.5E-08 | -0.042 (0.66) | 0.06 |
| Yes | 5135 (548/1607) |  | -0.017 (0.642) |  |
| Missing | 5125 (14/41) |  | -0.151 (0.773) |  |
| High cholesterol ever |  |  |  |  |
| No | 5259 (1038/3658) | 0.48 | -0.037 (0.674) | 0.56 |
| Yes | 5201 (232/791) |  | -0.026 (0.562) |  |
| Missing | 5457 (12/55) |  | -0.005 (0.613) |  |
| Cardiovascular disease ever |  |  |  |  |
| No | 5304 (991/3742) | 6.1E-09 | -0.024 (0.642) | 0.20 |
| Yes | 4965 (272/707) |  | -0.079 (0.71) |  |
| Missing | 5321 (19/55) |  | -0.178 (0.797) |  |
| Cancer ever |  |  |  |  |
| No | 5262 (1215/4327) | 0.003 | -0.035 (0.663) | 0.95 |
| Yes | 5046 (56/150) |  | -0.028 (0.401) |  |
| Missing | 4595 (11/27) |  | 0.039 (0.413) |  |
| *APOE-ε4* carrier |  |  |  |  |
| No | 5267 (945/3370) | 0.03 | 0.019 (0.521) | <2E-16 |
| Yes | 5204 (337/1134) |  | -0.191 (0.926) |  |

^a^Cox regression with adjustment for baseline age. Significance based on F-test

^b^Linear regression with adjustment for baseline age; only samples with at least three valid 3MSE scores were used for longitudinal analysis. Significance based on F-test

**Supplementary Table S5:** Number of participants and average Modified Mini-Mental State Exam scores at each visit year stratified by hormone therapy groups in WHIMS

| Visit Year | N | Mean ± SD | N | Mean ± SD | N | Mean ± SD | N | Mean ± SD |
| --- | --- | --- | --- | --- | --- | --- | --- | --- |
|  | **(E-alone-control)** | | **(E-alone)** | | **(E+P-control)** | | **(E+P)** | |
| 0 | 738 | 95.6 ± 3.9 | 708 | 95.4 ± 4.0 | 1532 | 96.2 ± 3.5 | 1495 | 96.1 ± 3.6 |
| 1 | 711 | 96.4 ± 3.2 | 669 | 96.1 ± 3.6 | 1468 | 96.7 ± 3.2 | 1431 | 96.6 ± 3.4 |
| 2 | 681 | 96.9 ± 3.19 | 631 | 96.4 ± 3.7 | 1427 | 97.0 ± 3.3 | 1395 | 96.9 ± 3.3 |
| 3 | 673 | 96.8 ± 3.3 | 642 | 96.6 ± 3.8 | 1418 | 97.2 ± 3.3 | 1386 | 96.9 ± 4.0 |
| 4 | 634 | 97.1 ± 3.6 | 625 | 96.6 ± 4.0 | 1346 | 97.3 ± 3.6 | 1326 | 96.9 ± 4.4 |
| 5 | 620 | 96.9 ± 4.1 | 599 | 96.4 ± 5.3 | 1305 | 97.0 ± 4.8 | 1268 | 96.9 ± 4.7 |
| 6 | 614 | 96.7 ± 3.9 | 579 | 96.3 ± 5.9 | 1268 | 97.1 ± 4.3 | 1242 | 96.9 ± 4.0 |
| 7 | 530 | 96.7 ± 4.1 | 493 | 96.4 ± 4.9 | 1080 | 97.0 ± 4.3 | 1053 | 97.0 ± 4.1 |
| 8 | 416 | 96.4 ± 4.7 | 371 | 96.4 ± 5.1 | 846 | 96.7 ± 4.6 | 805 | 96.7 ± 5.0 |
| 9 | 367 | 96.1 ± 5.5 | 345 | 96.4 ± 4.7 | 783 | 96.7 ± 4.6 | 756 | 96.3 ± 5.4 |
| 10 | 233 | 96.5 ± 6.1 | 224 | 96.5 ± 4.9 | 521 | 96.3 ± 5.4 | 493 | 96.6 ± 5.4 |
| 11 | 41 | 94.6 ± 12.2 | 35 | 97.0 ± 2.8 | 79 | 95.5 ± 9.3 | 84 | 96.3 ± 5.2 |

Notes: E = estrogen, P = progesterone
